# How do women feel cold water swimming affects their menstrual and perimenopausal symptoms?

**DOI:** 10.1177/20533691241227100

**Published:** 2024-01-25

**Authors:** Megan Pound, Heather Massey, Sasha Roseneil, Ruth Williamson, C Mark Harper, Mike Tipton, Jill Shawe, Malika Felton, Joyce C Harper

**Affiliations:** 1 EGA Institute for Women’s Health, 4919 University College London, London, UK; 2 School of Sport, Health and Exercise Science, 6697 University of Portsmouth, Portsmouth, UK; 3 1948 University of Sussex, Brighton, UK; 4 Hampshire Hospitals NHS Foundation Trust, Basingstoke, UK; 5 8721 University Hospitals Sussex NHS Foundation Trust, Worthing, UK; 6 Sørlandet Sykehus, Kristiansand, Norway; 7 6633 University of Plymouth and Royal Cornwall Hospitals NHS Trust, Cornwall, UK; 8 Department of Rehabilitation and Sport Sciences, 6657 Bournemouth University, Poole, UK

**Keywords:** Menopause, perimenopause, postmenopause, menstrual, cold water swimming

## Abstract

**Objective:**

This study aimed to determine how women felt cold water swimming affected their menstrual and perimenopausal symptoms.

**Study design:**

An online survey that asked women who regularly swim in cold water about their experiences. The survey was advertised for 2 months on social media. Questions related to cold water swimming habits and menstrual and perimenopausal symptoms were analysed.

**Main outcome measures:**

Quantitative and qualitative data including; frequency of menstrual and menopause symptoms, the effect of cold water swimming on these symptoms.

**Results:**

1114 women completed the survey. Women reported that cold water swimming reduced their menstrual symptoms, notably psychological symptoms such as anxiety (46.7%), mood swings (37.7%) and irritability (37.6%). Perimenopausal women reported a significant improvement in anxiety (46.9%), mood swings (34.5%), low mood (31.1%) and hot flushes (30.3%). The majority of women with symptoms swam specifically to reduce these symptoms (56.4% for period and 63.3% for perimenopause symptoms). Women said they felt it was the physical and mental effects of the cold water that helped their symptoms. For the free text question, five themes were identified: the calming and mood-boosting effect of the water, companionship and community, period improvements, an improvement in hot flushes and an overall health improvement.

**Conclusion:**

Women felt that cold water swimming had a positive overall effect on menstrual and perimenopause symptoms. Studies on other forms of exercise to relieve menstrual and perimenopause symptoms may show similar findings.

## Introduction

Women experience menstruation from puberty to menopause. Menstruation involves the shedding of the uterine lining approximately every 24–38 days and lasts up to 8 days.^1^ Puberty, or menarche, when the first period begin, usually happens around 12 years of age for girls in the United Kingdom (UK) but can start between 8 and 17 years.^2^

Women’s experience of their period varies greatly. For some, their quality of life (QoL) and wellbeing is significantly impacted by their period. Often, the initial period shapes perception and management throughout their lives – many have negative experiences around their menstrual cycle.^3,4^ In a recent study, we conducted using focus groups to ask year 10 (age 14–15 years) and perimenopausal women about their periods, and all those interviewed had negative feelings towards their period and the symptoms they encountered, with many perimenopausal women relating back to their puberty experiences.^5^

Menstruation symptoms, besides bleeding, include premenstrual syndrome (PMS), menstrual cramps (dysmenorrhoea), breast tenderness, mood changes, anxiety, depression, fatigue, bloating, headaches and food cravings, digestive cramps and acne and skin changes.^6^ Most of these changes are brought about through the cycling of the sex hormones, estrogen and progesterone. It is important to note that every woman’s experience of menstruation varies.

When a woman comes to the end of her reproductive life, her periods will stop. Menopause is defined as 12 months after the cessation of the last menstrual period and the woman is then considered postmenopausal.^7,8^ Perimenopause is the period before the official cessation of menstruation where fluctuating levels of estrogen and progesterone may cause women to experience perimenopausal symptoms.^9^ The menopause transition lasts on average 4 years with many women experiencing symptoms that significantly affect their QoL.^10,11^ Our studies have shown that peri- and postmenopausal women reported psychological symptoms more than any other.^12–14^

What should be classed as a perimenopausal symptom is controversial, but most would agree it includes changes in menstruation which leads eventually to cessation, vasomotor symptoms such as hot flushes and night sweats, genitourinary symptoms including changes in the vagina and psychological symptoms.^13,15^

Advice to women throughout their reproductive years is to balance their lifestyle with a healthy diet, exercise^16^ and good quality sleep.^17^ This may help relieve some of the menstrual and perimenopause symptoms.

The physical and mental benefits of swimming have been long established and the some of these benefits may be augmented when swimming in cold water,^18^ but we need more data from prospective studies to better investigate the short- and long-term health consequences of this important recreational activity. Ice baths or cold water immersion are frequently used to aid athletes’ recovery and muscle repair,^19^ but the actual benefits are debated.^20^ Increasingly, studies are suggesting that endocrine, cardiovascular and psychological benefits are derived from the adaptations developed through cold water swimming.^21,22^

A survey completed by Massey et al.^21^ showed the positive perceived impact on mental health conditions in individuals who swim outdoors; further case studies have indicated cold water swimming could be used as treatment for depression or major depressive disorder.^23^ Burlingham et al. published a feasibility trial on 59 patients with anxiety and/or depression which suggested that the activity brought benefits and that further study was justified.^24^ A controlled study by Massey et al. showed cold water swimming acutely improves mood and provides evidence suggesting a long-term improvement in novice cold water swimmers, although further research on the mechanism needs to be initiated.^25^ Furthermore, being present in an outside or ‘blue’ space has been shown to have a positive impact on mental health.^26^ It has been hypothesized the parasympathetic nervous system promotes this restoration, stimulating a stress recovery response.^27^

No research has focused specifically on the impact of cold water swimming on menstrual and perimenopausal symptoms, but current research can be applied to key menstrual and perimenopausal symptoms. Studies support the link between swimming and improved mental health.^28^ Weight bearing exercise is key to reduce the risk of osteoporosis, but swimming has been shown to improve the bone mineral density in women with osteoporosis.^29^ Cold water swimming triggers cutaneous vasoconstriction^18,30^ which may counteract the vasodilation causing the common perimenopausal symptom of hot flushes.

However, cold water swimming has risks such as hypothermia and, in extreme cases, death exacerbated by ‘cold shock’^31^ incapacitation and drowning^32^ and cardiac rhythm disturbances as a result of autonomic conflict.^33^ In addition, water quality standards can vary, with raw sewage pollution becoming an increasingly common concern in UK rivers and seas, increasing the likelihood of gastroenteritis and other infections.^34,35^

Using the current understanding of the physiological effects of regular cold water swimming, it is plausible that the activity could be of benefit for menstrual and perimenopausal symptoms. We therefore conducted a survey of women who regularly cold water swim to ask them how they felt swimming impacted these symptoms. The cold water swimming habits, including duration of swim, seasonal swimming, use of wetsuits and so on, amongst this population have recently been described.^36^

## Methods

This project was approved by UCL Research Ethics Committee: 9831/007. Participants provided informed consent to participate in this research.

### Research design

The design of the survey is detailed in Pound et al.^36^ In brief, a research team of academic and clinical experienced cold water swimmers (Joyce Harper, Sasha Roseneil, Ruth Williamson and Heather Massey) and designed the survey with advice from the public, mainly through local swimming groups. The survey was built on Qualtrics® and was delivered online, comprising of 42 questions, mostly multiple choice with some free-text questions (Supplementary Data 1).

The survey was validated through interviews with 6 women who were cold water swimmers. The final survey was advertised via cold water swimmers Facebook groups but was also promoted on social media. The survey was active from 7th June 2022 to 6th August 2022.

The survey was divided into four sections. Firstly, women were asked multiple-choice questions to provide demographic information, for example, describe their age, marital status and exercise regimes. The second section asked information on the participant’s cold water swimming habits. The third section asked about the participants’ experience of menstruation and/or the menopause, focussing on the effect cold water swimming has had on their symptoms. Finally, women were asked questions to determine the demographics of the survey participants such as religion and disability. All demographic questions included an option of ‘prefer not to say’ to ensure women did not feel pressured to answer these questions. The third section ended with a free text question, asking the participants to state anything about their views on the effects of cold water swimming on menstrual and menopause symptoms.

### Inclusion criteria

Participants were considered eligible if they were women who swim outdoors in unheated water.

### Data analysis

Stata was used to perform statistical hypothesis tests on the data. A Fisher’s exact test was used to calculate the significance values. Due to some of the data having very low observed frequencies and consequent low expected frequencies, a chi-squared test was considered unsuitable. Results were considered statistically significant if the *p*-value was less than 0.05. To reduce the probability of generating false significance, a lower *p*-value was considered for analysis of further subgroups. This was ultimately decided to be unnecessary. Hypothesis tests were performed to compare differing swimming habits with the five perimenopause symptoms and four menstrual symptoms most frequently stated to be reduced by cold water swimming.

Content analysis was used for the free text questions. The quotes were read and re-read to gain familiarization and initial codes were systematically distinguished, and relevant data was sorted into each code. This allowed for further familiarization with the data, as well as a chance to better recognize patterns. Repeated thoughts and particularly meaningful sentiments were noted alongside initial thoughts and ideas pertaining to the data. Codes were then sorted into themes that were reviewed against the overall dataset to ensure that it was representative.

## Results

### Demographics

1357 women started the survey and 1114 pressed submit (1114/1357, 82.1%). Only submitted responses were included in the study. The demographics are detailed in Pound et al.^36^

In brief, the overwhelming majority of the participants resided in the UK (1041/1114, 93.4%). The age of the participants ranged from 16 to 80 years, with a mean age of 49 years. Most of the participants were aged between 45 and 59 years (759/1114, 68.1%). Most of the participants were heterosexual (981/1114, 88.1%), and most were married/in a civil partnership (670/1114, 60.1%). Most had children (774/1114, 69.5%), but a large proportion did not (340/1114, 30.5%).

### Menstrual symptoms

**Figure 1.**
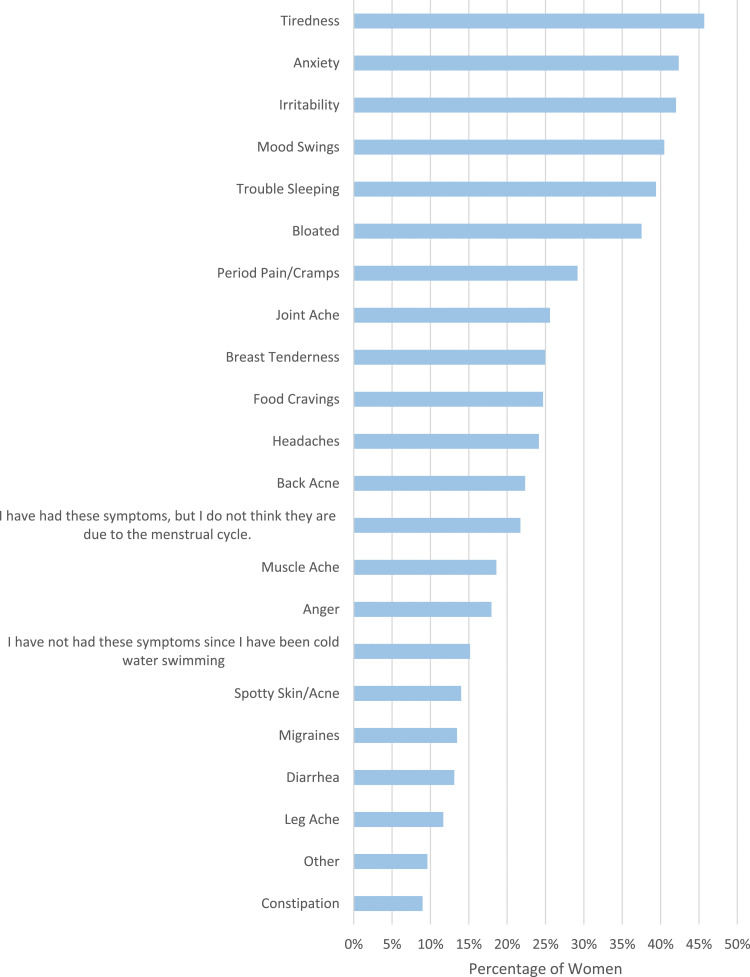
Women were asked to select symptoms they have experienced that they believe are due to their menstrual cycle, since they have been cold water swimming.

Women were asked to state menstrual symptoms they had experienced since they started cold water swimming (Figure 1). The most frequent were tiredness (509/1114, 45.7%), anxiety (472/1114, 42.4%), irritability (468/1114, 42.0%), mood swings (451/1114, 40.5%) and trouble sleeping (439/1114, 39.4%). Some women stated they had not experienced these symptoms since cold water swimming (169/1114, 15.2%) or stated they had experienced these symptoms, but did not think they are related to their menstrual cycle (242/1114, 21.7%). These women were not directed to the next questions.

**Figure 2.**
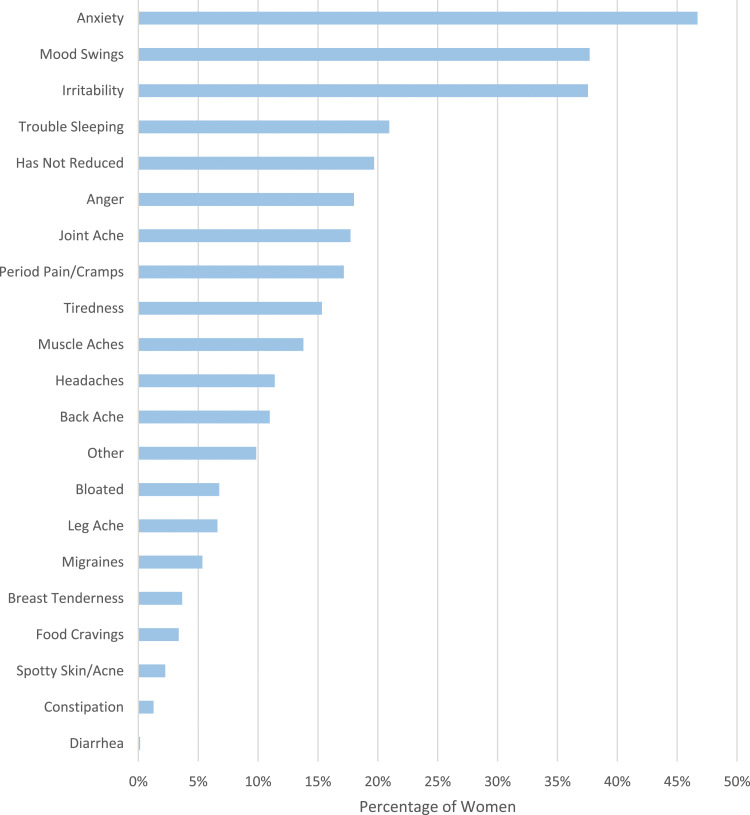
Women were asked to select menstrual cycle symptoms they believe have been reduced by cold water swimming.

The 711 women who stated they experienced menstrual symptoms since they had been cold water swimming were asked if they believed cold water swimming had reduced any of their menstrual cycle symptoms (Figure 2). Psychological symptoms were most commonly reported to be improved by cold water swimming: anxiety (333/711, 46.7%); mood swings (268/711, 37.7%) and irritability (267/711, 37.6%). Trouble sleeping was also commonly reported to be improved (149/711, 21.0%). If women selected their symptoms were not reduced by cold water swimming, they were not directed to the next question (140/711, 19.7%).

**Figure 3.**
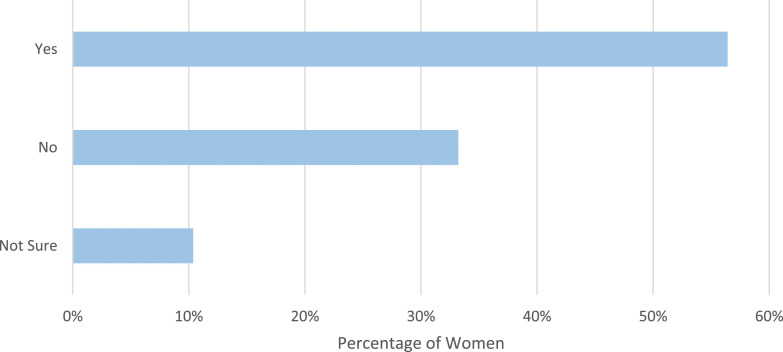
Women were asked if they swim specifically to relieve menstrual symptoms.

Women who stated a reduction in one or more menstrual symptoms were then asked if they swim specifically to reduce their menstrual symptoms (Figure 3). In total, 321 of these women (56.4%) stated they swim specifically to reduce these symptoms.

**Figure 4.**
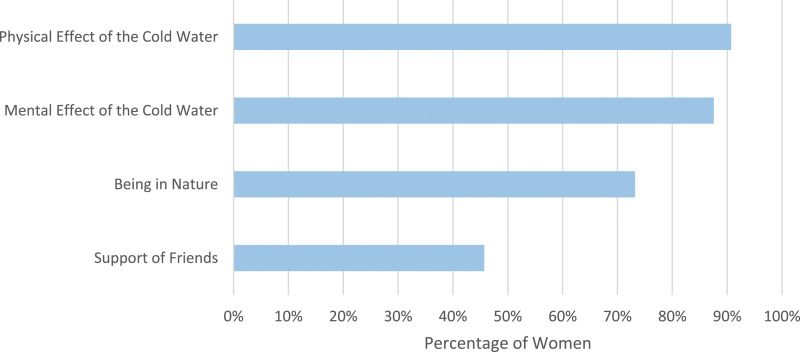
Women were asked why they believe cold water swimming reduces their menstrual symptoms.

Women were asked why they believe cold water swimming reduces their menstrual symptoms (Figure 4). The majority of women stated the physical effect (518/571, 90.7%) and the mental effect (500/571, 87.6%) of the cold water reduced their menstrual symptoms. Support of friends was less commonly selected (261/571, 45.7%).

**Figure 5.**
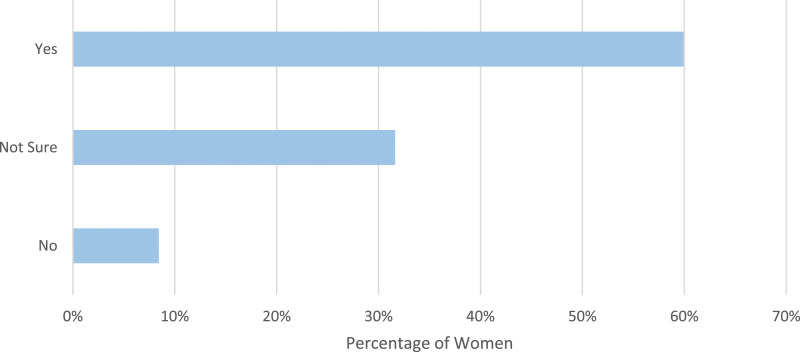
Women were asked if they feel the effect of the cold water is increased when the water is colder.

Women were then asked if they feel the effect of the cold water is increased when the water is colder (Figure 5). The majority of women stated yes (341/569, 59.9%).

Women who have experienced menstrual symptoms since cold water swimming were asked if they feel cold water swimming has increased any of their menstrual symptoms. The majority (622/710, 87.6%) stated cold water swimming has not increased their menstrual symptoms. 61 women stated ‘other’ (8.6%). Period pain/cramps (8/710, 1.1%), anxiety (8/710, 1.1%) and tiredness (8/710, 1.1%) were the most commonly reported symptoms to be increased by cold water swimming.

### Menopause symptoms

**Figure 6.**
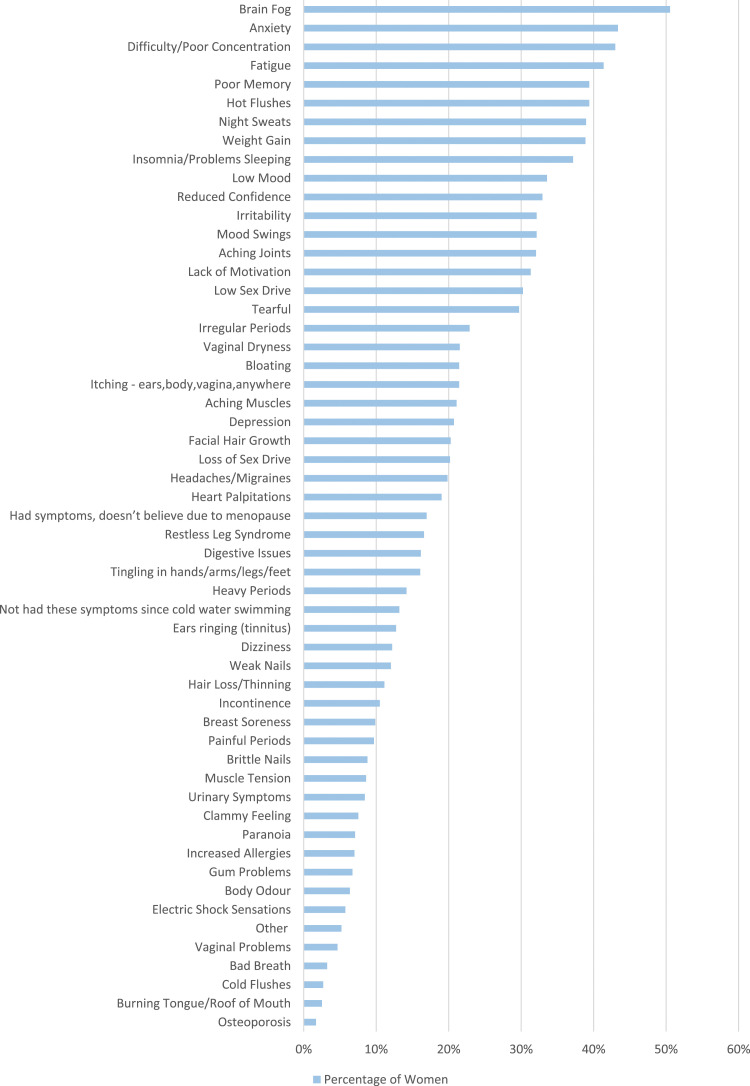
Women were asked to select symptoms they have experienced that they believe are due to the perimenopause, since they have been cold water swimming.

Women were asked to state the menopause symptoms they had experienced since they have been cold water swimming (Figure 6). Psychological symptoms such as brain fog (563/1114, 50.5%), anxiety (483/1114, 43.4%) and poor concentration (479/1114, 43.0%) were selected most frequently. Hot flushes (439/1114, 39.4%) and night sweats (434/1114, 39.0%) were also frequently selected. Some women stated they have not experienced these symptoms since cold water swimming (142/1114, 13.2%) or stated they had experienced these symptoms, but do not think they are related to the menopause (189/1114, 17.0%). These women were not directed to the next question.

**Figure 7.**
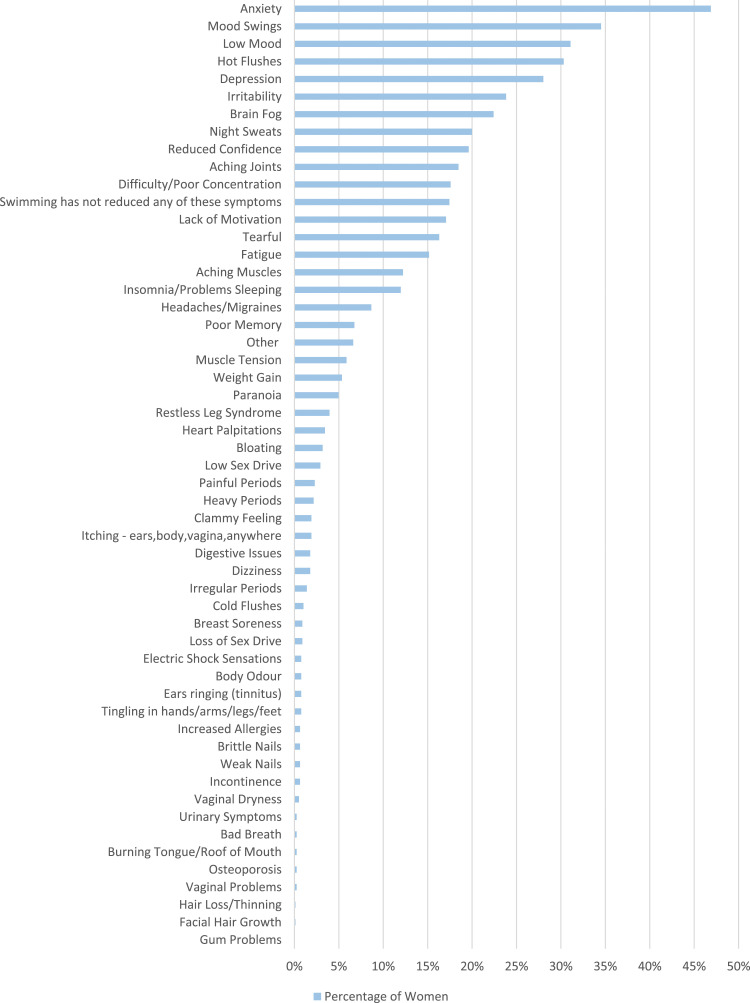
Women were asked if they felt cold water swimming had reduced any of their menopause symptoms.

Women who said they had experienced perimenopausal symptoms since cold water swimming were asked if they believe cold water swimming reduced any of their menopause symptoms (Figure 7). Psychological symptoms were reported to be the most affected by cold water swimming. In total, 46.9% of women (368/785) stated their anxiety was reduced by cold water swimming, followed by mood swings (271/785, 34.5%), low mood (244/785, 31.1%) and depression (220/785, 28.0%). Hot flushes (238/785, 30.3%) and night sweats (157/785, 20.0%) were also reported to be considerably reduced. A total of 137 women (17.5%) stated swimming had not reduced any of their menopause symptoms.

**Figure 8.**
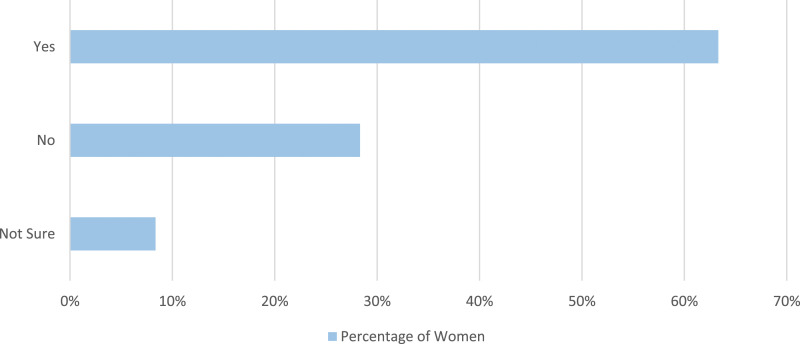
Women who had perimenopause symptoms were asked if they swim specifically to relieve their menopause symptoms.

Women who had perimenopause symptoms were asked if they swim specifically to relieve menopause symptoms. Of these women, 63.3% (409/646) stated they swim specifically to relieve these menopause symptoms (Figure 8).

**Figure 9.**
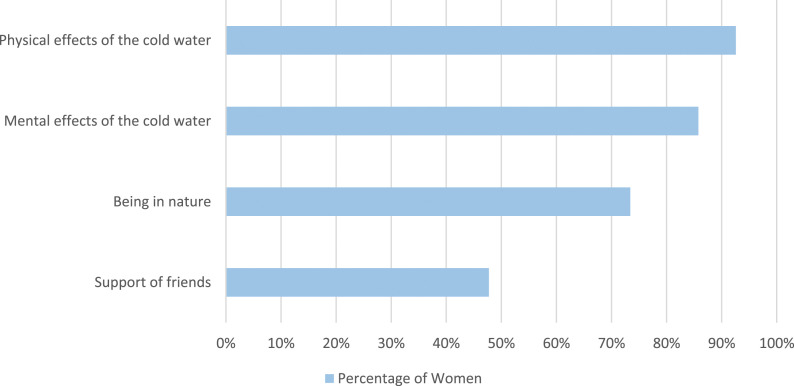
Women who have perimenopause symptoms were asked why they think cold water swimming relieves their menopause symptoms.

These women were asked why they think cold water swimming relieves their symptoms (Figure 9). Overwhelmingly, the physical effect of the cold water (559/599, 92.6%) and the mental effect of the cold water (555/599, 85.8%) were selected.

**Figure 10.**
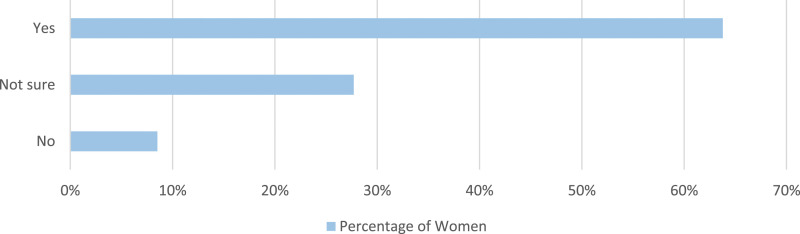
Women were asked if the effects are more pronounced when the water is colder.

Women were asked if the effects of the cold water on their symptoms are more pronounced if the water is colder (Figure 10). The majority stated yes (412/646, 63.8%).

Women were asked the reverse: stating if cold water swimming had increased any of their menopause symptoms. Most stated cold water had not increased any of their symptoms (715/785, 91.1%); however, 1% (8/785) of women thought hot flushes were worsened by cold water swimming.

### Swimming habits effects on symptoms

Details of the participants’ swimming habits are detailed in Pound et al. (2023). Here these data are compared to reports of their menstrual and perimenopausal symptoms.

### Menstrual data

A total of 1109 women stated they swim in the summer. Women who swim once a week compared to everyday were significantly more likely to report a reduction in their anxiety (81/230, 35.2%; 21/92, 22.8%; *p* = .034). There was no correlation between swimming more regularly in winter and a reported reduction in the 4 most commonly reported symptoms to be reduced: anxiety, mood swings, irritability and trouble sleeping.

Women who swam for 15–30 minutes in winter compared to <5 minutes were more likely to report a reduction in the 4 most commonly reported symptoms, although the results were not found to be significant.

Women who wore a wetsuit in winter and women who wore a wetsuit in the summer compared to women who wore a swimming costume in both seasons were more likely to report a reduction in these symptoms. Women were significantly more likely to report a reduction in their trouble sleeping if they wore a wetsuit in winter compared to a swimming costume (49/250, 19.6%; 93/739, 12.6%; *p* = .009).

### Perimenopause data

Women who swim every day in summer (92/1109, 8.3%) compared to once a week (230/1109, 20.7%) were significantly more likely to report a reduction in their hot flushes (35/92, 38.0%; 40/230, 17.4%; *p* = .0). Reduction in psychological symptoms (anxiety, low mood and mood swings) was observed when women swim more regularly in summer, although the results were not statistically significant (*p* = .272; *p* = .053; *p* = .191). Women who swim in winter a few times a week compared to a few times a month were significantly more likely to report a reduction in both anxiety (149/417, 35.6%; 34/134, 25.4%; *p* = .035) and mood swings (119/417, 28.5%; 22/134, 16.4%; *p* = .006).

Women who mostly swim for 15–30 minutes in winter compared to women that mostly swim for less than 5 minutes were significantly more likely to report an alleviation of hot flushes (89/342, 26.0%; 7/64, 10.9%; *p* = .01). They were also significantly more likely to select that cold water swimming improved their anxiety (126/342, 36.8%; 15/64, 23.4%; *p* = .045) and low mood (87/342, 25.4%; 8/51, 15.7%; *p* = .024), the two most commonly reported symptoms reduced by cold water swimming in this survey. They were more likely to report an alleviation of mood swings (94/342, 27.5%; 10/64, 15.6%), but this result was not significant (*p* = .06).

Women who wore swimming costumes/skins swimming in winter reported less alleviation of their psychological symptoms: anxiety (253/739, 34.2%; 95/250, 38.0%; *p* = .284), mood swings (184/739, 24.9%; 70/250, 28.0%; *p* = .357) and low mood (176/739, 23.8%; 65/250, 26.0%; *p* = .496), compared to women who stated they wear a wetsuit in winter but these results were not statistically significant.

In concordance with the menstrual symptom data, women who wore a swimming costume/skins compared to wetsuits in summer reported more reduction in anxiety (348/1037, 33.6%; 34/110, 30.9%; *p* = .597) and low mood (234/1037, 22.6%; 18/110, 16.4%; *p* = .147) but these results were not statistically significant. However, women who swim in swimming costume/skins compared to wetsuits in the summer were significantly more likely to report a reduction in their hot flushes (225/1037, 21.7%; 15/110, 13.6%; *p* = .049).

### Qualitative comments about menstrual and perimenopause symptoms and cold water swimming

In total, 742/1114 (66.6%) of women answered the question asking if they had any further comments about the effects of cold water on their menstrual and perimenopause symptoms.

From this emerged five key themes: (1) the calming and mood-boosting effect of the water, (2) companionship and community, (3) an improvement in hot flushes, (4) period improvements and (5) an overall health improvement.

#### Calming and mood-boosting effect of the water

Most of the responses detailed the mood-lifting effect of outdoor swimming, stating the waters anxiety relieving, relaxing and empowering properties.

‘Being outdoors and swimming has really helped my mental health.’ Age 48

‘It’s helped my anxiety and loss of confidence greatly. Helps me feel strong and capable when the world seems to imply I’m “past it.”’ Age 56

A large proportion of the women who felt the mood-boosting properties reasoned it was as a direct result of the cold water. One woman commented on the ‘dramatic’ difference in her mental health when swimming in a warm country for half of the year, and the UK for the other half.

‘I feel calmer after a swim. In the water, especially when it’s colder I can feel my body physically slowing down.’ Age 50

‘I have found that cold water is an immediate stress/anxiety reliever. It is my 'go to’ activity to reset myself…a cold water dip will immediately reset my anxiety levels and is rejuvenating. The colder the water, the more satisfying it is!’ Age 56

#### Companionship and community

Some women attributed the mood-boosting and symptom relieving property of cold water to the companionship and ‘camaraderie’ they have found being part of a community of like-minded women.

‘Cold water swimming has had a profound effect on my menopausal symptoms. Exercising in nature, alone or with a group of other women is healing. The camaraderie, shared stories & laughter are part of the magic…’ Age 54

‘Swimming with other women who are at the same phase of life is wonderful. We can laugh and cry together and have emotional conversations that seem quite natural in the cold sea. Being in cold water is both energising and calming at the same time…but mostly, it’s about the camaraderie, and the endorphin boost that sets you up for the day!’ Age 48

‘I find meeting for a swim in the river with people who are becoming friends, really helps with my mental wellbeing. I feel part of something and the laughing and talking really helps brighten my spirits. I don’t feel so alone.’ Age 49

A few women commented on the body-positivity they have noticed in this community and are now more comfortable in their ageing bodies, contributing to better mental health overall.

‘This is not related to the chemistry of cold water swimming, but I did find that the community of middle-aged and older women I met through participating made me feel much more positive about ageing, and that the pleasure in the exercise and what my body was capable of made me feel more positive about my body, and much less self-conscious.’ Age 53

Some women also commented on how the friendly community has created a safe space for them to discuss symptoms previously considered taboo.

‘Where I swim it’s usually groups of women who swim, there’s not as many men. This offers a female friendly space where we can talk about these things with as much weight as we are able to lend or share as much/little as we want to safely and in a supportive environment. The social side to this should not be underestimated.’ Age 47

#### Improvement in hot flushes

Some women noted an improvement in their hot flushes or overall temperature regulation. Of these women, some noted the effect cold water swimming has for a period after they exit; however, some stated this occurs on a day-to-day basis.

‘It has definitely reduced my menopausal symptoms. The strongest ones being hot flushes, night sweats, body odour, anxiety, mood swings. The hot flushes and night sweats has pretty much disappeared.’ Age 47

‘I have noted that if I reduce the number of occasions I cold water swim, the number of hot flushes I experience increases.’ Age 51

‘Before I started cold water swimming I experienced hot flushes and night sweats, but they have ceased since I began cold water swimming regularly.’ Age 53

One woman disagreed, stating the cold water intensified her hot flushes.

‘Due to my body being conditioned to immersion in cold water, its “thermometer” is always set to be cold so I feel that I am hotter more frequently and more intensely. I work in a hot environment and so this seems to exacerbate the intensity the hot flushes even more.’ Age 42

#### Period improvements

Some women noted an improvement in their physical menstrual symptoms: bleeding and stomach cramps.

‘When I started cold water swimming three years ago I was still having periods and the cold certainly eased (numbed!) any stomach cramps and just being outside alone in nature and challenging myself physically made a huge difference to my mood. My Preference is to swim at sunrise and there are no words to describe the sense of serenity as you stop in the still sea and watch the sun appear over the horizon on a cold morning - I found it calmed my mind and any stress or anger would drift away into the water.’ Age 46

‘My period is less predictable within the days of blood loss. On the days when I swim in very cold water, and for longer periods of time if I’m training for long events, my period can be lighter, but then in other months if I don’t swim as much or for as long it is far heavier. This is more pronounced in winter.’ Age 45

Other women commented on the improvement in their mental health and well-being whilst on their period or experiencing menstrual symptoms.

‘Overall well-being is massively improved when I swim, regardless of my period… but now maybe I see there is a correlation between my reduced period symptoms and swimming!! Used to have really bad migraines and tiredness and I’ve not had that since I’ve started swimming… but that might be other factors too.’ Age 23

‘I think it helps my anxiety which is amplified when I’m due my period. I don’t think I have felt any negative benefits to swimming in cold water it’s only helped me.’ Age 40

#### Overall health improvement

Many women stated the life-affirming effect of cold water swimming has had on them. Some women stated they feel ‘more alive’, or that cold water swimming has been ‘life-changing’, providing a ‘reset’.

‘I swim in cold water because it makes me happy, which helps with most things in life.’ Age 62

‘I feel frustrated if I don’t get my cold water FIX - it has become a significant part of my life and one where I feel in control of my body and mind - especially in the winter in very cold water - this is great for my mental health and self-confidence.’ Age 50

‘Cold water is phenomenal. It has saved my life. In the water, I can do anything. All symptoms (physical and mental) disappear and I feel like me at my best.’ Age 57

## Discussion

This survey was designed to determine if women who cold water swim feel that swimming affects their menstrual and perimenopause symptoms. Women reported a reduction in many symptoms including anxiety, mood swings, irritability and trouble sleeping for those with menstrual symptoms, and anxiety, mood swings and hot flushes for those with perimenopause symptoms. The majority of women swim to relieve these symptoms and they felt that symptoms were helped by the physical and mental effects of the cold water which was more pronounced when it was colder. How often they swam, how long for and what they wore were also important. Through this, we hope to encourage further research as to the physiology of this response and, in light of the benefits our participants reported, increase the number of women partaking in this activity and increase awareness in this community to ensure women swim safely.

Other sports can also reduce these symptoms, and in this study, we did not compare cold water swimming to other sports. In a previous study of the women in our survey, we found that women’s swimming habits varied but most of the women were likely to swim in both summer and winter, wearing swimming costume/skins year-round and most swim for 30–60 minutes in the summer which reduces to 5–15 minutes in winter.^36^ The reasons for swimming are also varied but the majority do so to be outside, with improved mental health, exercise and relief of menopausal and menstrual symptoms also being important factors.

### Psychological symptoms caused by menstruation and perimenopause

Psychological symptoms are common during menstruation and perimenopause. The women reported a reduction of menstrual symptoms, notably the psychological symptoms: anxiety (51.1%), irritability (41.5%) and mood swings (41.3%) when cold water swimming. One limitation of the question was that we did not ask if the effects were whilst they were swimming, post swimming or both or how long the change in symptoms would last for.

For the perimenopausal women, psychological symptoms (anxiety (46.9%), moods swings (34.5%), low mood (31.1%) and depression (28%)) were most reported to be improved by cold water swimming. Women expressed clear beliefs on the ability of the cold water to reduce their anxiety and refresh their mood.

Reduction in anxiety associated with the perimenopause was highest in those that swim most regularly in both summer and winter. In the summer, the number of times the women stated they swim per week elicited a greater reduction in anxiety. In winter, swimming every day, a few times a week or once a week was found to be similar in the reported reduction in anxiety, but women were significantly more likely to report a reduction in anxiety if they swam once week compared to once a month (*p* = .035). This suggests there is an association between the regularity and routine of cold water swimming and reduced symptoms of anxiety. This is also reiterated in free text section, where some women stated if they do not swim regularly, they can feel the change in their mood and the increase in their stress and/or anxiety. Our findings are consistent with that of previous studies regarding psychological benefits and regular cold water exposure.^23,24,37,38^

When adjusted for time spent in the water in winter, women who swam for longer were significantly more likely to report a decrease in two of the three most commonly reported perimenopausal psychological symptoms: anxiety (*p* = .045) and low mood (*p* = .024). Women were also more likely to report a reduction in the most commonly reported menstrual psychological symptoms: anxiety, mood swings, irritability and trouble sleeping. These results show the length of time spent in the cold water is significant. In summer, women, on average, had to swim for longer to report a decrease in the same symptoms. This could be explained by sea temperatures in the UK, where most of the participants stated they are from, increasing by an average 15°C during the year.^39^ The evidence in the literature is conflicting, with some studies stating no benefit to staying in the water longer, with a significant increase in risk as the body temperature falls.^40,41^ Another study found significant changes in blood markers, and consequent theorized effects of these changes only occurred after a significant period of immersion.^42^ Our data did not align with the time periods indicated in these studies (10 minutes or fewer or more than an hour), instead suggesting a period of more than 15 minutes in winter, or 30 minutes in summer, to be the most effective length of time to spend in the water with the goal of reducing mental health symptoms. However, the temperature of the water was not reported by women; a 15 min immersion at 5°C is likely to cause body cooling related problems, so care must be taken and the timing adjusted according to the temperature.^43^ Safety studies conducted on long distance open water swims and triathletes have suggested when spending a prolonged period in the water below 18 degrees, a wetsuit should be worn^44^ but this might not apply to the demographic of women the complete this survey. In our study, women who swam for longer in winter were more likely to report wearing a full wetsuit to swim, reducing the risk of cold.

Physical exercise has been shown to help mental health during the menopause, with aquatic exercise shown to have a powerful reduction in mental health symptoms in the general population.^45,47^ It is not fully clear, due to the design of our study, how many women are exercising in the water versus how many women ‘cold plunge’.

Although 84.5% of women stated that they commonly swim with other people, and 47.8% stated the support of their friends is a key factor in the alleviation of their symptoms and community being identified as a key theme in the qualitative data, women who swim with others were not significantly more likely to report a reduction in anxiety, low mood or mood swings compared to those who swim alone. However, it is clear that women value the friendship and community they have found through this activity which is consistent with studies which have demonstrated that being part of an exercise group is protective against depression and that friendship is an additional protective factor for overall mental health.^48,49^ Furthermore, a feasibility study of cold water swimming for depression and anxiety found that the sense of community that developed over the course of the study was key to the women’s positive experience of the intervention.^24^

In contrast, the therapeutic act of being alone in wild spaces has been shown to increase the feeling of freedom and a sense of sensitivity to oneself, echoing the voices of the women who described the elating and freeing feeling they experience in the free-text question.^50,51^

The survey was advertised mainly through cold water swimming Facebook groups and therefore the data are likely skewed towards women who are more likely to swim with others. Women may have joined these groups out of a desire to meet others who cold water swim and therefore are more likely to value this aspect of the recreation. Women who swim alone may have been less exposed to our survey. One woman also reported a negative experience within one of these groups, citing feeling the group was ‘cliquey’ and that she was unwelcome as a newcomer. This may have contributed to some women choosing to swim alone.

While there is clearly a reported improvement in menopausal psychological symptoms, the root cause of this improvement cannot be fully evaluated due to the confounding factors in this observational survey.

Although this is the first study focussing on menstrual and perimenopausal symptoms and cold water swimming, this finding is supported by existing literature on cold water and/or swimming and mental health improvement.^28,46,52,53^ Our study supports a positive effect of cold water swimming on mental health. Multiple studies have shown higher rates of elevated mood, reduced stress and reduction in reported symptoms in outdoor swimmers, and three controlled studies finding a significant difference between the swimmers and the controls.^21,54,55^ The controlled study by Demori et al. provides a mechanistic support of our data, hypothesizing the stress reduction may be as a result of the habituation of the hypothalamus-pituitary axis, and a resultant decease in stress blood markers.^55^ In our study, 28% of women reported an improvement and no women stating it made their depression worse; this supports the conclusion of a previous case study, where cold water swimming was successfully used as a treatment for depression^23^ and a feasibility trial showing reductions in both anxiety and depression.^24^

### Reduction in vasomotor symptoms

Hot flushes were most commonly reported (85%), and reported to be alleviated (85%), by women aged 45–59. Only eight women stated their hot flushes were made worse by the cold water. Therefore, the anecdotal evidence for cold water swimming on the reduction of hot flushes is persuasive, and it appears that cold water swimming is very unlikely to make them worse. This was corroborated in the free-text answers, with women explicitly stating the improvement in their hot flushes as a result of cold water swimming. Due to this being the first survey assessing menopause symptoms and cold water, there is no existing evidence-base regarding the alleviation of hot flushes, although the mechanism is plausible.

In this study, women who swam more regularly in summer reported reductions in their hot flushes (*p* = .002). Also, 20% of women stated cold water swimming reduced their night sweats and insomnia/problem sleeping was reduced in 12%. At this point, we are not in a position to indicate if there is causation between their swimming activity and changes in their symptoms. Yet, dysregulation of the hypothalmic-pituitary adrenal axis (HPA) may contribute to hot flushes.^56^ It is possible that repeated exposure to cold water could result in improved regulation of the HPA axis. Reductions in plasma ATCH and cortisol were significantly lower when regularly immersed in cold water.^37^ Physical exercise has also been found to decrease cortisol levels and improve sleep quality in females.^57^ Consequently, cold water swimming as an exercise performed regularly in cold water may reduce the effects of hot flushes and improve perceived sleep quality via improved HPA regulation, but this hypothesis needs further investigation.

Swimming for more than 5 minutes in winter was also associated with significantly more women reporting a reduction in hot flushes (*p* = .01), indicating time spent in the cold water is important in relieving this symptom. ‘Cold plunging’ is becoming an increasingly popular activity but may not have the same effect as moving in the water. It again introduces the idea of exercise as an important factor in this recreation; multiple studies also finding a positive correlation between exercise and decreased severity of vasomotor symptoms and increased quality of life.^58,59^

The reduction of hot flushes by the cold water may also have contributed to an overall reduction in anxiety, with the SWAN Report stating vasomotor symptoms contributed the largest factor for menopausal anxiety.^60^ Of the women who reported a reduction in hot flushes in our survey, over half also reported a reduction in anxiety. The link between the physical and psychological symptoms of the menopause is clear, and cold water swimming may therefore act jointly, eliciting a greater overall effect. When asked which aspect of the cold water they believed reduced their symptoms, 92.6% of participants stated the physical, and 85.8% the mental effects, again indicating a substantial connection between the two categories of symptoms. A further study considering physical activity found an increase in neurotrophic factors, and an ultimate reduction in menopause symptoms.^61^ Overall, this produces a greater rationale for cold water swimming as an exercise type for the general alleviation of menopause symptoms.

### Genitourinary symptoms

GSM symptoms were not commonly selected by women to have improved with cold water swimming, with less than 7% of women stating an improvement. There is no mention of genitourinary symptoms in the free-text response, indicating this is not a symptom women expect to be improved by cold water swimming. In addition, there is little evidence for a mechanistic explanation of any improvement in these symptoms as a result of the cold water.

### Other symptoms

In the free-text symptoms, some women commented on the alleviation of their general ‘aches and pains’, with 18.5% reporting an improvement in their aching joints due to the cold water. The vast majority of these women swam for more than 5 minutes in both summer and winter, again reiterating the importance of the time spent in the cold water. Cryotherapy and ice baths have been extensively researched for the reduction of muscle and joint pain for application in professional sport and training, implemented into general practice following extensive exercise or sport injury.^62,63^ Although muscle recovery is not the primary goal of the women in our study, the principle of pain relief can be applied to the joint and muscle pain associated with the menopause. In addition, for recreational swimmers, aquatic exercise takes the pressure off the working joints by reducing the load; swimming allows women with existing joint pain to exercise with reduced pain, the cold water acting in-conjunction to relieve the pain they feel.^64^

## Study limitations

The limitations of this study must be recognized. First, as this is an observational study, there were no controls and so the data are biased as only women who cold water swim were included. It is likely that we would get similar results for women who do other sports in nature such as paddle boarding, kayaking and sports that were not so risky such as walking. By using an online survey to conduct our research, there is no way to verify whether the participants met our inclusion criteria, and therefore, the results may be misaligned with that of the true population. Furthermore, the use of an online survey introduces a sampling bias, where those without access to the Facebook groups or the social media sites used would not have been able to fill it in.^65^ In addition, those who did fill in the survey are likely to have a self-selection bias, where women were more likely to fill in our survey if they noticed an association between menopause symptoms and cold water swimming, compared to women who did not notice nor experience this connection, potentially skewing our results.^66^ Additionally, the associations made cannot suggest causality, some people feel more able to swim because they have fewer symptoms rather than swimming to relieve or reduce symptoms. The study is also limited by the demographics of the sample: mostly representative of white, highly educated women.

Due to the scope of the survey covering both menopausal and menstrual symptoms, some of the participants would not be considered menopausal, and others stated they are past the age of experiencing menopause symptoms. Although they were removed from the menopause analysis, their cold water swimming habits are included which may have skewed some of the data, although efforts were made to avoid this.

## Conclusion

This study demonstrates the anecdotal improvement in menopause and perimenopause symptoms already reported in the media on a large scale, suggesting cold water swimming does have menopause symptom-reducing properties.

Future research should focus in more detail on the frequency, duration, temperature and exposure needed to elicit a reduction in symptoms for menstrual and perimenopause symptoms in a more diverse population. Additionally, pregnancy is another key stage in women’s reproductive lives and the habits and impact of cold water swimming in pregnant women who already cold water swim should be examined.

Women’s swimming habits were varied overall. Women who reported a reduction of the most commonly reported symptoms were more likely to report longer swimming durations and or were more regular swimmers. Teaching women to swim safely and encouraging them to swim regularly may have a benefit on the debilitating symptoms associated with the perimenopause.

## Supplemental Material

Supplemental Material - How do women feel cold water swimming affects their menstrual and perimenopausal symptoms? Supplemental Material for How do women feel cold water swimming effects their menstrual and perimenopausal symptoms? by Megan Pound, Heather Massey, Sasha Roseneil, Ruth Williamson, C Mark Harper, Mike Tipton, Jill Shawe, Malika Felton and Joyce C Harper in Post Reproductive Health.
