# Supplementary material for: How do women feel cold water swimming affects their menstrual and perimenopausal symptoms?
Source: Post Reprod Health. 2024 Jan 25;30(1):11–27. doi: 10.1177/20533691241227100 (PMC10928965; doi:10.1177/20533691241227100)
Supplement: Supplemental Material - How do women feel cold water swimming affects their menstrual and perimenopausal symptoms? [file sj-pdf-1-min-10.1177_20533691241227100.pdf]

Supplementary material 1 and 2

1. Cold water swimming and menstrual cycle/menopausal symptoms

---

Start of Block: Consent

Q1 **UCL ethics committee approval: 9831/007**
Title of Study: An Investigation of the Impact of Cold Water Swimming on Menstrual and Menopausal Symptoms
Department: University College London (UCL) EGA Institute for Women's Health
Name and Contact Details of the Principal Researcher: Professor Joyce Harper. Institute for Women's Health, UCL Email: joyce.harper@ucl.ac.uk Telephone: 07880 795791.

This project is being conducted in collaboration with Professor Sasha Roseneil, University of Sussex, Dr Ruth Williamson, University Hospitals Dorset, and Dr Heather Massey, University of Portsmouth. Before you decide to take part - please read the information below.

1. Introduction. You have been invited to take part in a UCL, Institute for Women's Health research study being conducted to evaluate if unheated, outdoor swimming (cold water swimming) has beneficial impacts on menstrual and menopausal symptoms. Before you decide it is important for you to understand why the research is being done and what taking part will involve. Please take time to read the following information carefully and discuss it with others if you wish. If there is anything that is not clear, or if you would like more information, please contact joyce.harper@ucl.ac.uk.

2. What is the project's purpose? Many women experience menstrual and menopause symptoms. In this survey we aim to find out what symptoms you have and evaluate if cold water swimming has any effect on these symptoms. As is routine when doing surveys, we will ask you some questions about your demographics, such as your religion, ethnicity, sexual orientation, and age so we can determine the type of women who have completed the survey. We will ask you some questions about your cold water swimming activities, and about your possible menstrual and menopause symptoms and if cold water swimming changes these symptoms.

3. Do I have to take part? It is up to you to decide whether or not to take part. If you do decide to take part you will be asked to answer the questions in the online survey which will take about 15 minutes to complete. You can withdraw at any time without giving a reason – simply do not submit your answers. If you decide to withdraw before completing the survey, your answers will not be used in the study. Once you have submitted your answers, we cannot withdraw them as the survey is anonymous.

4. What are the possible disadvantages and risks of taking part? The UCL Research Ethics Committee has approved this study, and we do not anticipate any risks to any individuals taking part in this study. After completing the survey, we will direct you to more information about the menopause. Some of the themes discussed may be uncomfortable or you may wish to seek help. The following organizations are able to provide extra advice and support: https://menopausesupport.co.uk/,
https://www.themenopausecharity.org/, https://www.menopausematters.co.uk/.

After completing the survey, we will direct you to a poster about menopause symptoms.
If you have any concerns about the answers to the questions in this survey relating to your health, you may wish to contact your doctor.

5. What are the possible benefits of taking part? There is no direct benefit from participating, however you may learn some information about the menstrual cycle and menopause.

6. What if something goes wrong? If you have any complaints regarding your treatment by the researchers, you can complain to Professor Harper on joyce.harper@ucl.ac.uk or to the Chair of UCL Research Ethics Committee on ethics@ucl.ac.uk. In the unlikely event of something serious occurring during or following your participation in the project, please also contact Professor Harper. However should you feel your complaint has not been handled to your satisfaction please contact the Chair of UCL Research Ethics Committee.

7. Will my taking part in this project be kept confidential? All the information that we collect about you during the course of the research will be kept strictly confidential. You will not be able to be identified in any ensuing reports or publications.

8. What will happen to the results of the research project? Following completion of the study, we aim to publish the results in a peer-reviewed journal and present the data at conferences and on social media. The anonymised data (that cannot be used to trace you) may be used by others for future research but no one will be able to identify you when this data is shared.

9. Local Data Protection Privacy Notice. The controller for this project will be University College London (UCL). The UCL Data Protection Officer provides oversight of UCL activities involving the processing of personal data task in the public interest, and can be contacted at data-protection@ucl.ac.uk. This 'local' privacy notice sets out the information that applies to this particular study. Further information on how UCL uses participant information can be found in our 'general' privacy notice (https://www.ucl.ac.uk/legal-services/privacy): The information that is required to be provided to participants under data protection legislation (GDPR and DPA 2018) is provided across both the 'local' and 'general' privacy notices. Your personal data will be processed so long as it is required for the research project. If you are concerned about how your personal data is being processed, or if you would like to contact us about your rights, please contact UCL in the first instance at data-protection@ucl.ac.uk. UCL's Data Protection Officer is Alex Potts - data-protection@ucl.ac.uk

Thank you for reading this information and for considering to take part in this research study. Your consent is important to us. Please can you click on the consent button below.

Q2 **Please read carefully:**

I am eligible because I swim outdoors in unheated water (cold water swimming)
I have read the above information and understand what the study involves
I understand that if I decide at any time that I no longer wish to take part in this project, I can withdraw immediately by not submitting my answers. Once I have submitted my answers I will be unable to withdraw them.

I understand that the information will be treated as strictly confidential and handled in accordance with the provisions of the General Data Protection Regulation , UK 2018.
I understand that my fully anonymised data may be shared with other researchers
I have received all the information that I require, have indicated yes to all the above statements and consent to take part in this study.

Consent: I meet the criteria and have read and agree with the statements above:

- ○ Consent (1)
- ○ I do not consent (2)

Q3 **There are four parts to the survey. We would like to ask you about yourself, your swimming experience, your experience of menstrual and/or menopause symptoms, and finally some basic information about who you are. It is really important that you complete all four parts as incomplete surveys cannot be included in our analysis. You will know when your survey results are submitted as you will receive more information about the menopause. Thank you.**

End of Block: Consent

Start of Block: Default Question Block

Page Break

Q4 **Please state your country of residence**

- ○ UK (4)
- ○ Other (5) ____

JS

Q5 **Please state your age in years:**

____

Q6 **Please state your sexual orientation.**

- ○ Heterosexual (1)
- ○ Lesbian/gay (2)
- ○ Bisexual (3)
- ○ Pansexual (4)
- ○ Asexual (5)
- ○ Prefer not to say (6)

Q7 **Please select the option that most reflects your current relationship status.**

- ○ Single (1)
- ○ In a relationship not cohabiting (2)
- ○ In a relationship cohabiting (3)
- ○ Married/civil partnership (4)
- ○ Widowed (9)
- ○ Prefer not to say (7)
- ○ Other - in your own words (8) ____

Q8 **Do you have children?**

- ○ 1 (1)
- ○ 2 (2)
- ○ 3 (3)
- ○ 4 or more (4)
- ○ I do not have children (5)
- ○ Prefer not to say (6)

Q9 **Generally, which apply to you? Tick all that apply.**

- ☐ I regularly exercise (accumulating at least 150 minutes of moderate exercise or 75 minutes of vigorous exercise in a week) (1)
- ☐ I sleep well (2)
- ☐ I eat a healthy diet most of the time/always (3)
- ☐ I am a moderate alcohol drinker (4)
- ☐ I do not drink alcohol at all (9)
- ☐ I do not smoke (5)
- ☐ I have good mental health (6)
- ☐ None of the above (8)
- ☐ Prefer not to say (7)

Q10 Besides cold water swimming, which other forms of exercise do you do? Tick all that apply

- ☐ Climbing (6)
- ☐ Cycling (9)
- ☐ Dancing (7)
- ☐ Fitness classes (3)
- ☐ Kayaking (5)
- ☐ Indoor, heated swimming (14)
- ☐ Paddle boarding (4)
- ☐ Racket sports (8)
- ☐ Running (1)
- ☐ Surfing (10)
- ☐ Skiing/snow boarding (11)
- ☐ Walking/hiking (15)
- ☐ Weight training (2)
- ☐ Yoga/pilates/tai chi/etc (12)
- ☐ Other (13) ____

Q11 **Part 2: We would like to ask you about your experience of cold water swimming.**

Q12 What are the main reasons you started cold water swimming? Tick all that apply.

- ☐ Exercise (1)
- ☐ Companionship (2)
- ☐ Being outside (3)
- ☐ Covid restrictions (4)
- ☐ Mental health (5)
- ☐ General health (7)
- ☐ Relieve menstrual symptoms (9)
- ☐ Relieve menopause symptoms (10)
- ☐ Relieve a specific health problem (8)
- ☐ Convenience (11)
- ☐ Cost (12)
- ☐ Other (6) ____

Q13 How long have you regularly been cold water swimming?

- ○ Just started (1)
- ○ Under a year (2)
- ○ 1-5 years (3)
- ○ Over 5 years (4)

Q14 Where do you usually cold water swim? Tick all that apply.

- ☐ Unheated, outdoor pool (1)
- ☐ Sea (3)
- ☐ Lake (4)
- ☐ Reservoir (5)
- ☐ River (6)
- ☐ Other (7) ____

Q15 Who do you usually swim with?

- ○ Alone (1)
- ○ One other person (5)
- ○ Small group (2)
- ○ Big group (3)
- ○ It varies with each swim (4)

Q16 Do you swim cold water swim in the summer and winter?

- ○ Summer only (1)
- ○ Winter only (2)
- ○ Both summer and winter (3)

Display This Question:

If Do you swim cold water swim in the summer and winter? = Summer only

Or Do you swim cold water swim in the summer and winter? = Both summer and winter

Q17 In the summer, approximately how often do you cold water swim?

- ◯ Every day (1)
- ◯ A few times a week (2)
- ◯ Once a week (3)
- ◯ A few times a month (4)
- ◯ Once a month (5)
- ◯ Once a year on holiday (7)
- ◯ Never (8)
- ◯ Other (6) ____

Display This Question:

If Do you swim cold water swim in the summer and winter? = Summer only

Or Do you swim cold water swim in the summer and winter? = Both summer and winter

Q18 In the summer, how would you describe most of your swims?

- ◯ Under 5 mins (6)
- ◯ 5-15 mins (1)
- ◯ 15-30 mins (2)
- ◯ 30-60 mins (7)
- ◯ Over an hour (3)
- ◯ I do not swim in the summer (5)

Display This Question:

If Do you swim cold water swim in the summer and winter? = Summer only

Or Do you swim cold water swim in the summer and winter? = Both summer and winter

Q19 In the summer, what do you usually wear to cold water swim? Tick all that apply.

- ☐ Swimming costume/skins (1)
- ☐ Rash vest, other layers (2)
- ☐ Shortie wet suit (3)
- ☐ Full wet suit (4)
- ☐ Neoprene gloves and socks (6)
- ☐ Swimming cap (7)
- ☐ Warm hat (9)
- ☐ Naked (8)
- ☐ I do not swim in the summer (5)

Display This Question:

If Do you swim cold water swim in the summer and winter? = Winter only

Or Do you swim cold water swim in the summer and winter? = Both summer and winter

Q20 In the winter, approximately how often do you cold water swim?

- ○ Every day (1)
- ○ A few times a week (2)
- ○ Once a week (3)
- ○ A few times a month (4)
- ○ Once a month (5)
- ○ Once a year on holiday (7)
- ○ Never (8)
- ○ Other (6) ____

Display This Question:

If Do you swim cold water swim in the summer and winter? = Winter only

Or Do you swim cold water swim in the summer and winter? = Both summer and winter

Q21 In the winter, how would you describe most of your swims?

- ○ Under 5 mins (6)
- ○ 5-15 mins (1)
- ○ 15-30 mins (2)
- ○ 30-60 mins (7)
- ○ Over an hour (3)
- ○ I do not swim in the winter (5)

Display This Question:

If Do you swim cold water swim in the summer and winter? = Winter only

Or Do you swim cold water swim in the summer and winter? = Both summer and winter

Q22 In the winter, what do you usually wear to cold water swim? Tick all that apply.

- ☐ Swimming costume/skins (1)
- ☐ Rash vest, other layers (2)
- ☐ Shortie wet suie (3)
- ☐ Full wet suit (4)
- ☐ Neoprene gloves and socks (6)
- ☐ Swimming cap (7)
- ☐ Warm hat (9)
- ☐ Naked (8)
- ☐ I do not swim in the winter (5)

Q23 **Part 3: We would like to ask you about your menstrual cycle and/or menopause symptoms since you have been cold water swimming.**

Q24 The following symptoms may be caused by the menstrual cycle. Since you have been cold water swimming, are you having, or have you had, any of the symptoms below which you feel are due to your menstrual cycle? Tick all that apply.

- ☐ Mood swings (2)
- ☐ Anger (3)
- ☐ Anxiety (4)
- ☐ Irritability (5)
- ☐ Headaches (6)
- ☐ Bloated (7)
- ☐ Breast tenderness (8)
- ☐ Food cravings (9)
- ☐ Migraines (10)
- ☐ Period pain/cramps (12)
- ☐ Spotty skin/acne (13)
- ☐ Back ache (14)
- ☐ Leg ache (15)
- ☐ Muscle ache (16)
- ☐ Joint ache (17)
- ☐ Diarrhea (18)
- ☐ Constipation (19)
- ☐ Trouble sleeping (21)
- ☐ Tiredness (22)

☐ Other (20) ____

☐ I have had these symptoms but I do not think they are due to my menstrual cycle (1)

☐ I have not had these symptoms since I have been cold water swimming (23)

Skip To: Q30 If The following symptoms may be caused by the menstrual cycle. Since you have been cold water swimm... = I have had these symptoms but I do not think they are due to my menstrual cycle

Skip To: Q30 If The following symptoms may be caused by the menstrual cycle. Since you have been cold water swimm... = I have not had these symptoms since I have been cold water swimming

Q25 Do you feel that cold water swimming has reduced any of your menstrual cycle symptoms? If yes, tick all that apply.

- ☐ Swimming has not reduced any of these symptoms (24)
- ☐ Mood swings (2)
- ☐ Anger (3)
- ☐ Anxiety (4)
- ☐ Irritability (5)
- ☐ Headaches (6)
- ☐ Bloated (7)
- ☐ Breast tenderness (8)
- ☐ Food cravings (9)
- ☐ Migraines (10)
- ☐ Period pain/cramps (12)
- ☐ Spotty skin/acne (13)
- ☐ Back ache (14)
- ☐ Leg ache (15)
- ☐ Muscle ache (16)
- ☐ Joint ache (17)
- ☐ Diarrhea (18)
- ☐ Constipation (19)
- ☐ Trouble sleeping (21)

- [ ] Tiredness (22)
- [ ] Other (20) ____

Skip To: Q29 If Do you feel that cold water swimming has reduced any of your menstrual cycle symptoms? If yes, ti... = Swimming has not reduced any of these symptoms

---

Q26 Do you sometimes go swimming specifically to relieve these symptoms?

- ○ Yes (1)
- ○ No (2)
- ○ Not sure (3)

Q27 Why do you think cold water swimming relieves your symptoms. Tick all that apply.

- [ ] Support of friends (1)
- [ ] Being in nature (2)
- [ ] The physical effects of the cold water (3)
- [ ] The mental effects of the cold water (4)

Q28 Are the effects more pronounced when the water is colder?

- ○ Yes (1)
- ○ No (2)
- ○ Not sure (3)

Q29 Do you feel that cold water swimming has increased any of your menstrual cycle symptoms? If yes, tick all that apply.

- ☐ Swimming has not increased any of these symptoms (23)
- ☐ Mood swings (2)
- ☐ Anger (3)
- ☐ Anxiety (4)
- ☐ Irritability (5)
- ☐ Headaches (6)
- ☐ Bloated (7)
- ☐ Breast tenderness (8)
- ☐ Food cravings (9)
- ☐ Migraines (10)
- ☐ Period pain/cramps (12)
- ☐ Spotty skin/acne (13)
- ☐ Back ache (14)
- ☐ Leg ache (15)
- ☐ Muscle ache (16)
- ☐ Joint ache (17)
- ☐ Diarrhea (18)
- ☐ Constipation (19)
- ☐ Trouble sleeping (21)

☐ Tiredness (22)

☐ Other (20) ____

Q30 **The following symptoms may be caused by the menopause. Since you have been cold water swimming, are you having, or have you had, any of the symptoms below which you feel are due to the menopause? Tick all that apply.**

- ☐ Hot flushes (12)
- ☐ Night sweats (1)
- ☐ Irregular periods (2)
- ☐ Heavy periods (32)
- ☐ Painful periods (33)
- ☐ Mood swings (7)
- ☐ Depression (27)
- ☐ Anxiety (21)
- ☐ Paranoia (23)
- ☐ Brain fog (30)
- ☐ Difficulty/poor concentration (46)
- ☐ Headaches/migraines (19)
- ☐ Poor memory (14)
- ☐ Tearful (36)
- ☐ Irritability (58)
- ☐ Low mood (31)
- ☐ Reduced confidence (54)
- ☐ Dizziness (15)
- ☐ Insomnia/problems sleeping (20)

- [ ] Fatigue (8)
- [ ] Lack of motivation (53)
- [ ] Low sex drive (45)
- [ ] Loss of sex drive (3)
- [ ] Vaginal dryness (4)
- [ ] Vaginal problems (5)
- [ ] Itching - ears, body, vagina, anywhere (6)
- [ ] Tingling in hands/arms/legs/feet (29)
- [ ] Clammy feeling (44)
- [ ] Heart palpitations (24)
- [ ] Weight gain (16)
- [ ] Bloating (41)
- [ ] Digestive issues (48)
- [ ] Osteoporosis (35)
- [ ] Aching joints (17)
- [ ] Aching muscles (18)
- [ ] Muscle tension (47)
- [ ] Restless leg syndrome (28)
- [ ] Burning tongue/roof of mouth (25)
- [ ] Gum problems (40)

- [ ] Bad Breath (51)
- [ ] Ears ringing (tinnitus) (52)
- [ ] Facial hair growth (49)
- [ ] Hair loss/thinning (59)
- [ ] Body odour (39)
- [ ] Incontinence (37)
- [ ] Urinary symptoms (55)
- [ ] Breast soreness (38)
- [ ] Weak nails (57)
- [ ] Brittle nails (42)
- [ ] Cold Flushes (43)
- [ ] Electric shock sensations (50)
- [ ] Increased allergies (56)
- [ ] Other - please state in your own words (34) ____
- [ ] I have some of these symptoms but I do not think they are due to the menopause (61)
- [ ] I have not had any of these symptoms since I have been cold water swimming (26)

Skip To: Q36 If The following symptoms may be caused by the menopause. Since you have been cold water swimming,... = I have not had any of these symptoms since I have been cold water swimming

Skip To: Q36 If The following symptoms may be caused by the menopause. Since you have been cold water swimming,... = I have some of these symptoms but I do not think they are due to the menopause

*

Q31 Do you feel that cold water swimming has reduced any of your menopause symptoms? If yes, tick all that apply.

- ☐ Swimming has not reduced any of these symptoms (61)
- ☐ Hot flushes (12)
- ☐ Night sweats (1)
- ☐ Irregular periods (2)
- ☐ Heavy periods (32)
- ☐ Painful periods (33)
- ☐ Mood swings (7)
- ☐ Depression (27)
- ☐ Anxiety (21)
- ☐ Paranoia (23)
- ☐ Brain fog (30)
- ☐ Difficulty/poor concentration (46)
- ☐ Headaches/migraines (19)
- ☐ Poor memory (14)
- ☐ Tearful (36)
- ☐ Irritability (58)
- ☐ Low mood (31)
- ☐ Reduced confidence (54)
- ☐ Dizziness (15)

- [ ] Insomnia/problems sleeping (20)
- [ ] Fatigue (8)
- [ ] Lack of motivation (53)
- [ ] Low sex drive (45)
- [ ] Loss of sex drive (3)
- [ ] Vaginal dryness (4)
- [ ] Vaginal problems (5)
- [ ] Itching - ears, body, vagina, anywhere (6)
- [ ] Tingling in hands/arms/legs/feet (29)
- [ ] Clammy feeling (44)
- [ ] Heart palpitations (24)
- [ ] Weight gain (16)
- [ ] Bloating (41)
- [ ] Digestive issues (48)
- [ ] Osteoporosis (35)
- [ ] Aching joints (17)
- [ ] Aching muscles (18)
- [ ] Muscle tension (47)
- [ ] Restless leg syndrome (28)
- [ ] Burning tongue/roof of mouth (25)

- [ ] Gum problems (40)
- [ ] Bad Breath (51)
- [ ] Ears ringing (tinnitus) (52)
- [ ] Facial hair growth (49)
- [ ] Hair loss/thinning (59)
- [ ] Body odour (39)
- [ ] Incontinence (37)
- [ ] Urinary symptoms (55)
- [ ] Breast soreness (38)
- [ ] Weak nails (57)
- [ ] Brittle nails (42)
- [ ] Cold Flushes (43)
- [ ] Electric shock sensations (50)
- [ ] Increased allergies (56)
- [ ] Other - please state in your own words (34) ____

Skip To: Q35 If Do you feel that cold water swimming has reduced any of your menopause symptoms? If yes, tick all... = Swimming has not reduced any of these symptoms

Q32 Do you sometimes go swimming specifically to relieve these symptoms?

- Yes (1)
- No (2)
- Not sure (3)

Q33 Why do you think cold water swimming relieves your symptoms. Tick all that apply.

- Support of friends (1)
- Being in nature (2)
- The physical effects of the cold water (3)
- The mental effects of the cold water (4)

Q34 Are the effects more pronounced when the water is colder?

- Yes (1)
- No (2)
- Not sure (3)

Q35 Do you feel that cold water swimming has increased any of your menopause symptoms? If yes, tick all that apply.

- ☐ Swimming has not increased any of these symptoms (61)
- ☐ Hot flushes (12)
- ☐ Night sweats (1)
- ☐ Irregular periods (2)
- ☐ Heavy periods (32)
- ☐ Painful periods (33)
- ☐ Mood swings (7)
- ☐ Depression (27)
- ☐ Anxiety (21)
- ☐ Paranoia (23)
- ☐ Brain fog (30)
- ☐ Difficulty/poor concentration (46)
- ☐ Headaches/migraines (19)
- ☐ Poor memory (14)
- ☐ Tearful (36)
- ☐ Irritability (58)
- ☐ Low mood (31)
- ☐ Reduced confidence (54)
- ☐ Dizziness (15)

- Insomnia/problems sleeping (20)
- Fatigue (8)
- Lack of motivation (53)
- Low sex drive (45)
- Loss of sex drive (3)
- Vaginal dryness (4)
- Vaginal problems (5)
- Itching - ears, body, vagina, anywhere (6)
- Tingling in hands/arms/legs/feet (29)
- Clammy feeling (44)
- Heart palpitations (24)
- Weight gain (16)
- Bloating (41)
- Digestive issues (48)
- Osteoporosis (35)
- Aching joints (17)
- Aching muscles (18)
- Muscle tension (47)
- Restless leg syndrome (28)
- Burning tongue/roof of mouth (25)

- Gum problems (40)
- Bad Breath (51)
- Ears ringing (tinnitus) (52)
- Facial hair growth (49)
- Hair loss/thinning (59)
- Body odour (39)
- Incontinence (37)
- Urinary symptoms (55)
- Breast soreness (38)
- Weak nails (57)
- Brittle nails (42)
- Cold Flushes (43)
- Electric shock sensations (50)
- Increased allergies (56)
- Other - please state in your own words (34) ____

Q36 **In your own words, would you like to tell us anything about your views on the effects of cold water swimming on menstrual and menopausal symptoms.**

____

____

____

Q37 **Part 4: Finally, we need to know a little about your background so we can compare different groups of people. It is really important that you complete this section, as otherwise we cannot use your answers. You will know when the survey is submitted as you will receive information about the menopause.**

Page Break

Q38 **What is your highest educational qualification?**

- ○ Secondary School (1)
- ○ A Level/College-level (6)
- ○ University undergraduate (2)
- ○ University postgraduate (3)
- ○ Other (4) ____
- ○ Prefer not to say (5)

Q39 **What is/was your field of study/work/trade?**

____

Q40 **What is your religion or belief:**

○ No religion or belief (1)

○ Christian including Church of England, Catholic, Protestant and all other Christian denominations (2)

○ Hindu (3)

○ Jewish (4)

○ Muslim (5)

○ Sikh (6)

○ Buddhist (7)

○ Any other religion or belief - please give details (8) ____

○ Prefer not to say (9)

Q41 **How do you identify yourself? Choose one or more.**

- ☐ White - English / Welsh / Scottish / Northern Irish / British (1)
- ☐ White - Irish (2)
- ☐ Any other White background (please specify) (3) ____
- ☐ Black/Black British - African (4)
- ☐ Black/Black British - Caribbean (10)
- ☐ Any other Black/African/Caribbean background (please specify) (11) ____
- ☐ Latino (16)
- ☐ Asian/Asian British - Indian (12)
- ☐ Asian/Asian British - Pakistani (13)
- ☐ Any other Asian background (please specify) (5) ____
- ☐ Arab (8)
- ☐ Mixed ethnic background (please specify) (7) ____
- ☐ Any other ethnic group, please describe (9) ____
- ☐ Prefer not to say (15)

Q42 **What is your disability status?** The Equality Act 2010 states a person has a disability if they have a physical or mental impairment that has a substantial and long-term adverse effect (likely to last 12 months or more) on their ability to perform normal day-to-day activities (e.g. eating, washing, walking and going shopping).

- ☐ No disability (1)
- ☐ Sensory impaired (2)
- ☐ Physical or mobility impaired (3)
- ☐ Specific learning difficulty or disability (e.g. dyslexia) (4)
- ☐ General learning disability (cognitive) (5)
- ☐ Long term illness or health condition (6)
- ☐ Autistic spectrum disorder (7)
- ☐ Other, please specify (8) ____
- ☐ Prefer not to say (9)

Page Break

Q43 You will now be directed to the Menopause Poster produced by Pausitivity which will tell you more about some of the menopause symptoms.

Thank you for your time in completing our survey. We value and appreciate your participation. If you would like to discuss any of the issues that came up in this questionnaire, please know that there are sources of support available. In the first instance, you may wish to contact your doctor. Samaritans - https://www.samaritans.org/how-we-can-help/- An organization dedicated to providing support when experiencing distress or worries.

End of Block: Default Question Block

Start of Block: Block 2
